# Modeling mortality risk in patients with severe COVID-19 from Mexico

**DOI:** 10.3389/fmed.2023.1187288

**Published:** 2023-05-26

**Authors:** Arturo Cortes-Telles, Esperanza Figueroa-Hurtado, Diana Lizbeth Ortiz-Farias, Gerald Stanley Zavorsky

**Affiliations:** ^1^Respiratory and Thoracic Surgery Unit, Hospital Regional de Alta Especialidad de la Peninsula de Yucatan, Yucatan, Mexico; ^2^Department of Physiology and Membrane Biology, University of California, Davis, CA, United States

**Keywords:** COVID-19, disparities, Mexico, modeling, mortality, prediction, SARS-CoV-2, underserved

## Abstract

**Background:**

Severe acute respiratory syndrome caused by a coronavirus (SARS-CoV-2) is responsible for the COVID-19 disease pandemic that began in Wuhan, China, in December 2019. Since then, nearly seven million deaths have occurred worldwide due to COVID-19. Mexicans are especially vulnerable to the COVID-19 pandemic as Mexico has nearly the worst observed case-fatality ratio (4.5%). As Mexican Latinos represent a vulnerable population, this study aimed to determine significant predictors of mortality in Mexicans with COVID-19 who were admitted to a large acute care hospital.

**Methods:**

In this observational, cross-sectional study, 247 adult patients participated. These patients were consecutively admitted to a third-level referral center in Yucatan, Mexico, from March 1st, 2020, to August 31st, 2020, with COVID-19-related symptoms. Lasso logistic and binary logistic regression were used to identify clinical predictors of death.

**Results:**

After a hospital stay of about eight days, 146 (60%) patients were discharged; however, 40% died by the twelfth day (on average) after hospital admission. Out of 22 possible predictors, five crucial predictors of death were found, ranked by the most to least important: (1) needing to be placed on a mechanical ventilator, (2) reduced platelet concentration at admission, (3) increased derived neutrophil to lymphocyte ratio, (4) increased age, and (5) reduced pulse oximetry saturation at admission. The model revealed that these five variables shared ~83% variance in outcome.

**Conclusion:**

Of the 247 Mexican Latinos patients admitted with COVID-19, 40% died 12  days after admission. The patients’ need for mechanical ventilation (due to severe illness) was the most important predictor of mortality, as it increased the odds of death by nearly 200-fold.

## Introduction

Severe acute respiratory syndrome caused by a coronavirus (SARS-CoV-2) is responsible for the COVID-19 disease pandemic that began in Wuhan, China, in December 2019 (1). Since then, nearly seven million deaths have occurred worldwide due to COVID-19 (2). Mexicans are especially vulnerable to the COVID-19 pandemic as Mexico has the worst observed case-fatality ratio in the world (4.5%) (3). Since Hispanics represent a vulnerable population, factors predicting mortality must be understood in this ethnic group.

Older age, lower income, race/ethnicity, the need for invasive mechanical ventilation, HIV status, metabolic acidosis, and acute respiratory distress syndrome have consistently been related to worse outcomes in patients admitted to general wards, including other Hispanic populations (4–7). Compared to white patients, Mexican Latinos are more likely to have COVID-19-associated hospitalizations and admissions into the intensive care unit (ICU) and have a higher relative risk of death due to COVID-19 (8). Beyond demographic and some clinical biomarker parameters focused on identifying risk factors for mortality, other biomarkers are rarely reported in Hispanic populations. Thus, examining additional clinical biomarkers in whole blood may enhance outcome predictability.

In the Mexican population, available information used to develop predictive models has primarily considered demographic variables from a public database (9, 10), including cases not identified by the severity of the disease. Therefore, selection bias of this information might exist, and the generalizability of results may not be guaranteed. Since Mexican Latinos represent a vulnerable population, this study aimed to determine predictors of death in Mexicans with COVID-19 who were admitted to a large general acute care hospital using LASSO regression to identify demographic variables and routine care biomarkers that can predict risk factors of mortality. Based on some recent reports that age, several blood parameters, and the need for invasive mechanical ventilation are predictive of outcomes in various patient populations afflicted with COVID-19 (11–18), this study aimed at modeling predictors of mortality in patients with previous Severe COVID-19. We hypothesized that some of these predictors would also be prognostic in Mexican Latinos. Specifically, we hypothesized that age, sex, pulse oximetry saturation, and neutrophil count at admission would predict mortality.

## Materials and methods

### Study design

This was an observational cross-sectional study from the High Specialty Regional Hospital of Yucatan, Mexico, from March 2020 to August 2020. We consecutively enrolled 250 patients with confirmed COVID-19 during the period. Inclusion criteria were adults over 18 years old admitted to the hospital. To be admitted to the hospital, patients must be persistently short of breath, especially those whose pulse oximetry value was ≤94% on room air or who had symptoms of persistent cough, chest tightness, dizziness, and confusion (19). The High Specialty Regional Hospital Ethics Committee in Yucatan, Mexico, approved this study (Protocol number 2020–024). All patients provided written informed consent to participate in this study in compliance with the Helsinki Declaration and the Hospital Ethics Committee. Data were obtained and used from the admitted patient’s electronic medical record and recorded in password-protected computers.

### Statistical analysis

Continuous variables are expressed as mean (SD), and categorical variables are expressed as absolute values and percentages. A Mann–Whitney *U* test was used to compare age, pulse oximetry saturation at admission, days of hospital stay, and all the peripheral venous blood variables between patients alive at discharge and patients dying in the hospital. A comparison of proportions was used to compare the male/female ratio, those needing IMV, those with acute respiratory distress syndrome (ARDS) at admission, those with organ failure at admission, obesity, self-reported diabetes, and hypertension between these groups. A two-proportion z-test was also used to compare the proportion of patients with abnormal values for various blood variables between groups. Reference values for neutrophil and lymphocyte counts and the neutrophil-to-lymphocyte ratio were obtained from Forget et al. (20). Reference values for leukocyte, eosinophils, and platelet counts were from Wakeman et al. (21). Reference values for the platelet-to-lymphocyte ratio were from Wu et al. (22). High C-reactive protein values were defined as >3.0 mg/L (23). High D-dimer values were defined as >500 μg/L for patients ≤50 years of age and age-adjusted cut-off value by multiplying the patient’s age by 10 for those >50 years of age (24). A high derived neutrophil to lymphocyte ratio (dNLR) was defined as ≥2.6 (25). Several variables were outside the normal reference range between the two groups: patients alive at discharge compared to patients dying at the hospital. To account for multiple comparisons, the Benjamini-Hochberg procedure was used to control the false discovery rate (26), which we set to 0.05.

The least absolute shrinkage and selection operator (LASSO) regression was first used to identify possible predictors of mortality outcome. As there were several possible predictors of mortality outcome, LASSO regression (logistic) was used to select the most important predictor variables while minimizing prediction errors and overfitting. *K*-fold cross-validation (10-fold) was used to improve model performance.

The independent variables (predictors) used in LASSO regression were (1) age, (2) sex, (3) intubated and receiving invasive mechanical ventilation (1 = yes, 0 = no), (4) number of days on the mechanical ventilator, (5) length of hospital stay before being discharged or dying (days), (6) pulse oximetry saturation on admission (SpO_2_) (%), (7) whether the patients had acute respiratory distress syndrome (ARDS) on admission (1 = yes, 0 = no), (8) organ failure on failure on admission (1 = yes, 0 = no), (9) history of hypertension (1 = yes, 0 = no), (10) history of diabetes (1 = yes, 0 = no), (11) obesity (defined as a body mass index ≥30 kg/m^2^, determined by physician visual inspection), (12) the admission ward where the patient was first placed [1 = intensive care unit (ICU), 0 = somewhere in hospital as there was no space in the ICU], (13) leukocyte concentration at admission, (14) neutrophil concentration at admission, (15) lymphocyte concentration at admission, (16) eosinophil concentration at admission, (17) C-reactive protein (CRP) at admission, (18) D-dimer (DDIMER) concentration on admission, (19) platelet concentration at admission, (20) neutrophil to lymphocyte ratio (NLR) on admission (21) derived neutrophil-to-lymphocyte ratio at admission (dNLR, defined as the absolute neutrophil count divided by the difference between leukocytes and neutrophils) (27, 28), and (22) platelet to lymphocyte ratio at admission. Since the blood parameters were not normally distributed [per Kolmogorov–Smirnov test of normality (29)] and were substantially positively skewed, the data of the leukocyte concentration, neutrophil concentration, lymphocyte concentration, NLR, dNLR, eosinophil concentration, D-dimer concentration, and platelet count at admission were transformed by taking the logarithm of the values [Log_10_(X + 1)]. For the baseline CRP concentration, the values were not normally distributed; they were moderately positively skewed, so the data was transformed by taking the square root of its concentration.

Once the LASSO logistic regression identified the most important predictors of mortality outcome, binary logistic regression was used to develop several models based on the LASSO-identified predictors. Binary logistic regression was performed using the backward: likelihood ratio method. This stepwise method enters all independent variables at once. Then it removes each variable one at a time according to the probability of the likelihood-ratio statistic until only the significant variables remain in the model. Several models were compared via the Bayesian Information Criterion (BIC) for fit. Models with a lower Bayesian Information Criterion (BIC) fit better than those with higher BICs (30). All independent predictors were also evaluated via the variance inflation factor (VIF) (31) to control for multicollinearity. A VIF of 1 indicates no multicollinearity, while values >1 indicate some multicollinearity. For example, suppose the VIF for a predictor variable is 2.0. In that case, it means that the variance of the coefficient for that predictor (i.e., its standard error) in the full model is twice as large as the variance of the coefficient in the model with just that predictor; or in other words, twice as large than would be the case with no collinearity effect (31). A variance inflation factor ≥ 2.5 indicates considerable collinearity (31). However, to be conservative, it was decided that any predictor from a model with a VIF ≥ 2.0 would be eliminated.

Goodness-of-fit for binary logistic models was assessed using the Hosmer-Lemeshow test (32). This test compares the predicted probabilities of the logistic model to the observed outcomes of the data and assesses whether the model is a good fit for the data. The sample is divided into predefined bins or groups based on the model’s predicted probabilities to perform the test. The observed and expected frequencies in each bin are then compared using a chi-square test statistic. If the model fits the data well, the observed and expected frequencies should be similar, the test statistic will be small, and the *value of p* will be >0.05. However, if the test statistic is large, it suggests that the model is not a good fit for the data and *p* < 0.05. When the Hosmer-Lemeshow test for a given model candidate was statistically significant (*p* < 0.05), the model was not used for further evaluation. Any case with a standardized residual ≥2.5 was removed from the final model. For any missing data, multiple imputations were performed.

All data were analyzed by IBM SPSS Statistics (Version 29, Chicago IL) and R (33). The “*glmnet*” R package Version 4.1–6 obtained LASSO regression and binary logistic results (34, 35), and the BIC was obtained from “*AICcmodavg*” R package version 2.3–1. A *value of p* of <0.05 was used to signify statistical significance.

## Results

After removing outliers, 247 subjects ranging from 21 to 94 years of age (mean, 54 years old) were included in the analysis. Approximately one-third of the patients were female. Other variables are presented in Table 1. The number and percentage of patients outside the normal reference range are presented in Table 2.

**Table 1:** Patient characteristics of those afflicted with COVID-19.

	Patients that were alive at discharge (*n* = 146)	Patients that ended up dying at the hospital (*n* = 101)	Combined Total (*n* = 247)
Sex	95 M; 51F	72 M; 29F	167 M; 80F
Age (yrs)	49 (14) [47–52]^*^	61 (14) [25–83]	54 (15) [52, 56]
Was mechanical ventilation ever Needed?	Yes = 15 ^*^ No = 131	Yes = 94 No = 7	Yes = 109 No = 138
Days on mechanical ventilation	1.6 (6.2) [0.8–2.6]^*^	9.2 (7.5) [7.8–10.7]	4.7 (7.7) [3.8–5.7]
Hospital stay (days)	7.6 (8.0) [6.4–9.0]^*^	12.3 (8.7) [10.6–14.2]	9.5 (8.6) [8.5–10.7]
Pulse oximetry saturation at admission (%)	88% (7%) [87–89%]^*^	72% (19%) [68–75%]	81% (15%) [79–84%]
Acute Respiratory Distress Syndrome (ARDS) at admission	Yes = 45* No = 101	Yes = 75 No = 26	Yes = 109 No = 138
Organ failure on admission	Yes = 8* No = 138	Yes = 27 No = 74	Yes = 35 No = 212
History of hypertension	Yes = 44 No = 102	Yes = 39 No = 62	Yes = 83 No = 164
Self-reported Type II Diabetes	Yes = 38 No = 108	Yes = 38 No = 63	Yes = 76 No = 171
Obesity (BMI ≥30 kg/m^2^)	Yes = 74 No = 72	Yes = 55 No = 46	Yes =129 No = 118
Site of admission	Hospital (no space in ICU) = 132 ICU = 4	Hospital (no space in ICU) = 69 ICU = 32	Hospital (no space in ICU) = 201 ICU = 36
Leukocyte concentration at admission (cells per mm^3^)	10,552^*^ [9,830–11,349]	14,743 [13,475–16,017]	12,266 [11,552–12,969]
Neutrophil concentration at admission (cells per mm^3^)	8,441^*^ [7,672–9,224]	12,195 [11,792–14,197]	10,271 [9,548–10,971]
Lymphocyte concentration at admission (cells per mm^3^)	1,251^*^ [1,151–1,354]	1,054 [933–1,192]	1,170 [1,100–1,241]
Neutrophil to Lymphocyte Ratio at admission	8.8^*^ [7.7–10.0]	16.3 [14.2–18.5]	11.9 [10.6–13.2]
Derived neutrophil to lymphocyte ratio	4.7^*^ [4.1–5.2]	8.4 [7.5–9.4]	6.2 [5.6–6.7]
Platelet concentration at admission (x 10^9^ platelets per L of blood)	321 [294–352]	260 [260–300]	305 [288–324]
Platelet to lymphocyte ratio at admission	318 [284–355]	364 [313–419]	337 [306–373]
Eosinophil concentration at admission (number of cells per μL of blood)	65^*^ [49–83]	37 [20–57]	53 [41–66]
C-Reactive Protein concentration at admission (mg/L)	145^*^ [128–162]	220 [192–247]	175 [161–191]
D-Dimer concentration at admission (ng/mL)	1,256^*^ [953–1,637]	3,103 [2,203–4,007]	2,004 [1,596–2,492]

Data are shown as mean. [Brackets] represent the 95% bias-corrected and accelerated (BCa) Bootstrapped CI for the mean. The asterisk ^*^ signifies that the difference between groups was statistically significant at a false discovery rate of 5% when adjusted for multiple testing (22 tests). The derived neutrophil to lymphocyte ratio is neutrophils ÷ (leukocytes – neutrophils).

**Table 2:** The number and percentage of subjects outside of the normal reference range.

	Patients that were alive at discharge (*n* = 146)	Patients that ended up dying at the hospital (*n* = 101)	Combined total (*n* = 247)
Number & percentage of patients whose pulse oximetry saturation at admission was <90%	79 (54%)^*^	82 (81%)	161 (85%)
Number & percentage of patients whose Leukocyte concentration at admission was outside of the normal reference range (< 3,600 or > 9,200 cells per mm^3^) (21)	76 (52%)^*^	84 (83%)	160 (65%)
Number & percentage of patients whose Neutrophil concentration at admission was outside of the normal reference range (<1,800 or 6,900 cells per mm^3^) (20)	85 (58%)^*^	86 (85%)	171 (69%)
Number & percentage of patients whose neutrophil to lymphocyte ratio (NLR) at admission was outside of the normal reference range (<0.8 or > 3.7) (20)	113 (77%)^*^	95 (94%)	208 (84%)
Number & percentage of patients whose derived neutrophil to lymphocyte ratio (dNLR) at admission was clinically high (≥ 2.6) (25)	103 (71%)^*^	90 (89%)	193 (78%)
Number & percentage of patients whose lymphocyte concentration at admission was outside of the normal reference range (<1,300 or 3,800 cells per mm^3^) (20)	90 (62%)	76 (75%)	166 (67%)
Number & percentage of patients whose platelet concentration at admission was outside of the normal reference range (< 140 or > 320 × 10^9^ platelets per L of blood for males; and < 180 or > 380 × 10^9^ platelets per L of blood for females) (21)	55 (38%)	50 (50%)	105 (43%)
Number & percentage of patients whose platelet to lymphocyte ratio (PLR) at admission was outside of the normal reference range (< 36.6 or > 149.1 for males; and < 43.4 or > 172.7 for females) (22)	115 (79%)	78 (77%)	193 (78%)
Number & percentage of patients whose Eosinophil concentration at admission was clinically high (> 400 cells per μL of blood) (21).	4 (3%)	3 (3%)	7 (3%)
Number & percentage of patients whose C-Reactive Protein concentration at admission was clinically high (> 3 mg/L) (23).	145 (99%)	101 (100%)	246 (100%)
Number & percentage of patients whose D-Dimer concentration at admission was clinically high (> 260 ng/mL) (24).^#^	122 (84%)	88 (87%)	210 (85%)

Percentages were rounded to the nearest whole number for simplicity. ^#^ The D-Dimer value was missing for two patients alive at discharge and three patients dying in the hospital, so the number of subjects was 144 and 98, respectively. The asterisk ^*^ signifies that the difference in proportions between groups was statistically significant at a false discovery rate of 5% when adjusted for multiple testing.

After cross-validation, the LASSO logistic regression analysis revealed an overall model of six predictors plus the intercept (Supplementary Table S1). The best LASSO logistic model output is presented in Supplementary Table S2. LASSO logistic regression revealed six statistically important predictors of mortality outcome ranked by the most to least significant: (1) the patient was so ill that he/she needed to be placed on a mechanical ventilator, (2) logarithm of the platelet concentration at admission, (3) the logarithm of the derived neutrophil to lymphocyte ratio at admission, (4) the number of days of hospital stay, (5) age, and (6) pulse oximetry saturation at admission. A higher platelet concentration and pulse oximetry saturation at admission were protective against mortality and a shorter length of hospital stay. The mean binomial deviance was the smallest when lambda = 0.0129 (Supplementary Figure S1). However, since the length of hospital stay is not known at admission, leaving the variable in the model could be questioned as the length of stay is not known until later. Furthermore, when we used binary logistic regression on the same predictors as the LASSO model, multicollinearity was absent when the length of hospital stay was removed from the model. Indeed, the difference in BIC between the five and six predictor model was negligible (the difference in the BIC between the five and six predictor model was 1.1, which is negligible) (30).

As such, the binary logistic analysis revealed an overall model of five predictors that were statistically reliable in predicting which patients would be alive at discharge to which patients would die in the hospital after admission [−2 Log-Likelihood = 99.0, Nagelkerke R^2^ = 0.83; Omnibus tests of model coefficients χ^2^ = 235.2, d*f* = 5, *p* < 0.001, Bayesian Information Criterion (BIC) = 132.3]. The need to be placed on mechanical ventilation was the most significant predictor of mortality, followed by the logarithm of platelet concentration. The logarithm of the dNLR and age (years old) were the other positive predictors of mortality. On the other hand, higher SpO_2_ and platelet counts were protective against mortality (Table 3). The model was a good fit [Hosmer and Lemeshow Test, χ^2^ = 5.94, df = 8, *p* = 0.65] (Table 3). Other visual graphics of the model’s fit are presented in Supplementary Figures S2–S4 in the online supplement. The variance inflation factor was below 2.0 for all five predictors: Mechanical Ventilation = 1.81, the logarithm of the derived neutrophil to lymphocyte ratio = 1.17, age = 1.38; pulse oximetry saturation at admission = 1.05; logarithm of the platelet counts = 1.51.

**Table 3:** Results of the binary logistic regression analysis (*n* = 247 patients).

							95% CI for odds ratio
Variables	B	S.E.	Wald	*z*-value	Sig.	Odds ratio	Lower to Upper
Age (years) (Range = 21 to 94 years old)	0.073 [0.02, 0.14]	0.022	10.6	3.25	0.001	1.08	1.03 to 1.12
Is mechanical ventilation needed? (1 = yes, 0 = no)	5.26 [4.39, 7.82]	0.77	46.4	6.81	<0.001	193	43 to 878
Pulse oximetry saturation at admission (%) (Range = 20 to 100%)	−0.043 [−0.10, −0.01]	0.022	4.4	−2.01	0.044	0.96	0.92 to 0.99
The logarithm of dNLR at admission Log_10_(X + 1) [Range of Log10 (X+1)= 0.18 to 1.47]	2.65 [0.46, 6.70]	1.27	4.1	−2.09	0.037	14.1	1.2 to 169.5
The logarithm of platelets counts at admission Log_10_(X + 1) [Range of Log10 (X+1)= 1.46 to 3.26 x 10^9^ cells per L]	−6.41 [−11.70, −3.28]	2.02	10.0	−3.17	0.002	0.002	0.00003 to 0.09
Constant	9.53 [0.67, 19.76]	4.86	3.85	1.96	0.050		

Each independent variable had 1 degree of freedom, including the constant. Brackets represent the 95% bootstrapped CI for the coefficients. Bootstrapped results are based on 1,000 bootstrapped samples. Three outliers (1%) were removed (standardized residuals ≥ 2.5). The variance inflation factor was the following for each predictor: age = 1.37; pulse oximetry saturation at admission = 1.05; mechanical ventilation = 1.80; logarithm of the platelet counts = 1.51; logarithm of dNLR = 1.16. Bayesian Information Criterion (BIC) = 132, (Hosmer & Lemeshow Goodness-of-Fit = *χ*^2^ = 6.11, d_f_ = 8, *p* = 0.64), j – 2 log likelihood = 99.0, Nagelkerke’s pseudo *R*^2^ = 0.83. In the first column, the minimum and maximum values in the dataset are presented within parentheses. So the model is accurate between each of these ranges. The [Brackets] in the second column represent the 95% CI.

The model demonstrated that patients’ need for mechanical ventilation (due to severe illness) was the most important predictor of mortality, as it increased the odds of death by nearly 200-fold. Every 1% increase in pulse oximetry saturation upon admission decreased the odds of death by about 4%; for every one-year increase in age, the odds of death increased by 8%. In addition, for every 1 unit increase in the log of platelet concentration, the odds of death decreased by nearly 100%. For every 1 unit increase in the log of dNLR, the odds of death increased by 14-fold (Table 3).

## Discussion

The primary purpose of this study was to identify reliable predictors of mortality in adult Mexican Latinos with severe COVID-19 admitted to the hospital. These patients were admitted due to persistent shortness of breath or had symptoms of cough, chest tightness, dizziness, or confusion (19). The predictors of mortality from most to least important (but still statistically significant) were (1) being so sick that patients needed to be placed on a mechanical ventilator, (2) low platelet concentration at admission, (3) increased dNLR at admission, (4) increased age, and (5) decreased pulse oximetry saturation at admission. Increased hospital stay also predicted mortality; nevertheless, it was not included in the model as the length of stay is unknown at admission.

In general, patients that ended up dying in the hospital had more leukocytes and neutrophils, had a larger NLR and dNLR at admission, and had higher values for CRP and D-Dimer compared to those alive at discharge. Also, the prevalence of those with ARDS and systemic organ failure was higher in the group that ended up dying than those alive at discharge (Table 2). This demonstrates an association between mortality and morbidity outcomes for white blood cell parameters, ARDS, and the need for invasive mechanical ventilation, similarly found in Chinese patients afflicted with COVID-19 (11, 14, 15).

Published studies using the Mexican COVID-19 Epidemiologic Surveillance System demonstrated that being male, increased age, and having comorbidities such as diabetes, COPD, obesity, hypertension, and immunosuppression were associated with death (9, 10). In contrast, the current data found that hypertension, obesity, diabetes, and organ failure did not predict mortality.

A recent report has identified that requiring invasive mechanical ventilation does not predict mortality but rather the delay of intubation in patients with similar characteristics (36). This finding may represent our population since there was an ~8-day delay in assistance at the emergency department for evaluation after initial symptoms (37). Altogether, our results should strengthen the recommendation to seek prompt medical attention after a COVID-19 diagnosis.

The previous history of comorbidities has been associated with adverse outcomes, yet, the data usually have included patients with all disease severity ranges. In severe COVID-19, we found no difference in morbidities other than older age, as previously reported (10). Therefore, the value of our results is to be generalized in patients with severe COVID-19.

The binary logistic regression model was initially based on the response from the LASSO logits regression (Supplementary Tables S1, S2; Supplementary Figure S1). The model had an acceptable goodness-of-fit, and the normality assumption of residuals was met (Supplementary Figures S1–S3). It was determined that five important predictors (out of 22) best-predicted mortality outcomes. Of these five predictors, the top three most important predictors of mortality outcome – in order of influence – were: the need for invasive mechanical ventilation, low platelet count at admission, and increased dNLR at admission. All five predictors did not have significant confounding or multicollinearity. High pulse oximetry saturation values at admission and high platelet counts at admission were protective of mortality, while increases in age, dNLR, and the need for invasive mechanical ventilation increased the odds of death. These findings support the findings of Camacho-Moll et al. (38), whose final logistic model included respiratory and organ-injured routine biomarkers (38). Even though some studies have shown that CRP and D-dimer can be a prognosticator in morbidity and mortality in those with COVID-19 (13, 17, 18), the concentrations were similarly high in both groups, so they did not present as essential predictors in mortality outcome in this study.

Preliminary risk factors for mortality in the Mexican population have been described during the first wave of COVID-19 (37). The need for invasive mechanical ventilation and an NLR ≥ 9 significantly increased the odds of dying from COVID-19 (37). Subsequently, some studies involving the native Mexican Latino population have suggested cut-off values of NLR ranging from 5.43 (AUC 0.80) to >10.0 (OR 2.2) as predictors of poor outcome (39–42). We determined that dNLR was more predictive than NLR in the current study. While we did not have a cut-off in dNLR, every 1 unit increase in the log of dNLR increased the odds of death by 14-fold (Table 3). Therefore, a subsequent analysis could reinforce this finding and be applied in clinical practice.

A common practice of binary logistic regression is examining the fraction of correctly classified responses. Here, one chooses a cut-off on the predicted probability of positive responses (Usually at 50.0%) and then predicts that responses will be positive if the predicted probability exceeds this cut-off (43). If we did this using the binary logistic model in Table 3 and a cut-off of predicted probability of 50% and then predicting mortality outcome if the probability ≥50.0%, the area under the ROC (AUC) curve would be 0.912. Furthermore, the remaining classification results would be as follows: the true negative rate (specificity) = 90%, the true positive rate (sensitivity) = 92%, the false positive rate = 10%, the false negative rate = 8%, the false discovery rate = 13%, the false omission rate = 6%, positive predictive value = 87%, negative predictive value = 94%. Performing this type of 2 × 2 binary classification is frowned upon since a logistic model is a model for the probability of an event, not a model for the event’s occurrence (43). Second, the fraction of correctly classified responses highly depends on the cut-point chosen for a “positive” prediction (43). Third, one can add a highly significant variable to the model and decrease the percentage classified correctly (43). Classification error is insensitive and statistically inefficient (43). Fourth, if diseases were present in only 2% of the population, one could be 98% accurate in diagnosing the disease by ruling that everyone is disease free by avoiding predictors. The proportion classified correctly fails to consider the task’s difficulty (43). That is why the AUC is not a valid measure of a diagnostic / screening test (44). Therefore, the model presented in Table 3 is solely for prediction.

Since the beginning of the pandemic, researchers worldwide have set out to identify the factors related to adverse outcomes in patients requiring hospital admission due to unfavorable disease progression. Demographic factors, morbidities, clinical data, laboratory tests including routine biomarkers, and those tests challenging to implement routinely (including cytokines, and growth factors, among others) have been identified in the ordinary care of cases. With these data, risk calculators have been proposed and implemented in the emergency care room to recognize cases that could have an adverse outcome. These calculators include multiple demographic, laboratory, and imaging variables with various areas under the curve (AUC) of 0.75 to 0.88. Among these calculators: LUCAS (45), 4-C Mortality Index (46), NEWS2 (47), and COVID-GRAM (48) have been developed in different populations.

The four calculators mentioned above (44–47) have advantages and disadvantages in identifying cases that could potentially have adverse outcomes. However, the common denominator is that none included Mexican Latinos. Therefore, they lack external validity applicable to our population. Furthermore, as stated above, AUC is not a valid measure of a diagnostic / screening test (44). If a study wanted to model the occurrence of an event, using Mathew’s correlation coefficient would be a better classification statistic (41, 49, 50).

Also, another limitation of the previously mentioned calculators is that they come from the same population (NEWS2 versus COVID GRAM Italian population) and have not had the same predictive accuracy (47). COVID GRAM has been tested in a group of European Hispanics in Spain, and the results are not similar to the Chinese population (51). Specific data on outcomes in a Latino population are limited to some countries. Peruvian patients admitted with COVID-19 have identified age, oxygen saturation less than 80% at admission, use of ivermectin, azithromycin, and leukocyte levels on admission as risk factors for mortality (52–54), but in other Latino population data is scarce.

Some of the limitations of this study include the limited sample size and the fact that data was collected at a single hospital site. A multi-center study would have increased the sample size and the findings’ generalizability. However, in the Yucatan Peninsula, opportunities for collaboration are scarce, and the number of hospital centers in the region is lacking. Nevertheless, we bootstrapped the results in the logistic regression model to improve reliability and reduce sampling error. Thus, despite these limitations, the model is appropriate for the population studied.

## Conclusion

This paper presents one of the first statistical models used in the Mexican population to predict mortality outcomes. It was determined that the most important predictors of death resulting from severe COVID-19 illness were the need for invasive ventilation, reduced platelet counts, increased dNLR, increased age, and reduced pulse oximetry saturation, all measured at hospital admission. The model revealed that these five variables shared ~83% variance in outcome. Based on these findings, some potential future research directions include (1) validation of the identified preditors in large and more diverse populations, (2) investigation of the impact of interventions like immune modulators and mechanical ventilation strategies on mortality, and (3) and long-term follow-up on the survivors.

### Guarantor

AC-T and GZ are the guarantors of the manuscript’s content, including the data and analysis.

## Data availability statement

The original contributions presented in the study are included in the article/Supplementary material, further inquiries can be directed to the corresponding author.

## Ethics statement

The Ethics Committee of the High Specialty Regional Hospital in Yucatan, Mexico, approved this study (Protocol number 2020–024). All patients provided written informed consent to participate in this study in compliance with the Helsinki declaration and the Hospital Ethics Committee.

## Author contributions

AC-T, EF-H, and DO-F performed the literature review, designed the study, and collected and interpreted the data. GZ performed the statistical analysis, interpreted the data, and wrote the initial manuscript, and approved the final draft. AC-T edited the manuscript. All authors contributed to the article and approved the submitted version.

## Conflict of interest

The authors declare that the research was conducted in the absence of any commercial or financial relationships that could be construed as a potential conflict of interest.

## Publisher’s note

All claims expressed in this article are solely those of the authors and do not necessarily represent those of their affiliated organizations, or those of the publisher, the editors and the reviewers. Any product that may be evaluated in this article, or claim that may be made by its manufacturer, is not guaranteed or endorsed by the publisher.

## Abbreviations

SARS-CoV-2: Severe acute respiratory syndrome caused by a coronavirus
MX: Mexico
ARDS: acute respiratory distress syndrome
dNLR: neutrophil to lymphocyte ratio
LASSO: Least absolute shrinkage and selection operator
ICU: intensive care unit
CRP: C-reactive protein
NLR: neutrophil to lymphocyte ratio
BIC: Bayesian Information Criterion
VIF: variance inflation factor
AUC: area under the curve
