# Supplementary material for: Modeling mortality risk in patients with severe COVID-19 from Mexico
Source: Front Med (Lausanne). 2023 May 26;10:1187288. doi: 10.3389/fmed.2023.1187288 (PMC10263446; doi:10.3389/fmed.2023.1187288)
Supplement: Supplementary file 1 [file Data_Sheet_1.PDF]

ONLINE SUPPLEMENT

Modeling mortality risk in patients with severe COVID-19 from Mexico.

Arturo Cortes-Telles[1†] ORCID: **0000-0002-5322-5604**

Esperanza Figueroa-Hurtado[1] ORCID: **0000-0003-1388-0572**

Diana Lizbeth Ortiz-Farias[1] ORCID: **0000-0002-3212-1929**

Gerald Stanley Zavorsky[2†] ORCID: **0000-0002-4473-1601**

[1] Respiratory and Thoracic Surgery Unit. Hospital Regional de Alta Especialidad de la Peninsula de Yucatan. Yucatan, Mexico.

[2]Department of Physiology and Membrane Biology, University of California, Davis, United States of America.

[†]These authors share first authorship.

This supplement is peer-reviewed.

Supplementary Table S1. Full model output of the Lasso logistic regression with a binary outcome determined that six variables (plus the intercept) were the most parsimonious model, typically measured by cross-validation.

Variables	Full model output s1
Intercept	2.907
Age (years old)	0.026
Sex (0 = male; 1 = female)	0.0
Pulse oximetry saturation on admission (%)	-0.021
ARDS on admission (1 = yes; 0 = no)	0.0
Organ failure on admission (1 = yes, 0 = no)	0.0
Diabetes (1 = yes, 0 = no)	0.0
Obesity (1=yes, 0 = no)	0.0
Number of days on mechanical ventilation	0.0
Site of admission (1 = ICU, 0 = hospital)	0.0
Mechanical ventilation needed (1= yes; 0 = no)	3.498
Number of days of hospital stay	-0.004
The logarithm of baseline leukocytes at admission	0.0
The logarithm of baseline neutrophils at admission	0.0
The logarithm of baseline lymphocytes at admission	0.0
The logarithm of baseline neutrophil to lymphocyte ratio on admission	0.0
The logarithm of baseline eosinophils at admission	0.0
The logarithm of baseline platelet counts at admission	-1.01
The square root of the baseline c-reactive protein concentration at admission	0.0
The logarithm of the baseline D-Dimer at admission	0.0
The logarithm of baseline platelets to lymphocyte ratio at admission	0.0
The logarithm of baseline dNLR at admission	0.849

Supplementary Table S2. Best model output of the Lasso logistic regression with a binary outcome. The response depends on only six predictors. Being placed on mechanical ventilation was the most important predictor, followed by platelet counts at admission (second most important), dNLR at admission (third), number of days of hospital stay (fourth), age (fifth), and pulse oximetry saturation at admission (sixth).

**Variables**	**Best model output s0 (log odds)**	**Odds ratio**
Intercept	1.15	3.16
Mechanical ventilation needed (1= yes; 0 = no)	0.72	2.05
The logarithm of baseline platelet counts at admission	-0.19	0.82
The logarithm of baseline dNLR at admission	0.15	1.16
Number of days of hospital stay	-0.006	0.99
Age (years old)	0.0034	1.003
Pulse oximetry saturation on admission (%)	-0.0032	0.997

The intercept represents the estimated log odds of mortality outcome when all predictor variables are zero.

Supplementary Figure S1. Mean cross-validated error curve using ten-fold cross-validation on the data. Logistic regression is shown with the binomial response, specifically, binomial deviance (minus twice the log-likelihood on the left-out data) versus the log of lambda (λ). The top of each plot is annotated with the size of the models.[1] The two dotted vertical lines represent the one-standard-deviation band. The left vertical line corresponds to the minimal mean cross-validated error. In contrast, the right vertical line is the largest value of lambda such that the mean error is within one standard error of the minimum mean cross-validated error– the so-called one-standard-error" rule).[1] The lambda value that minimizes means the squared error is 0.01289. That is the base of the natural logarithm, $e^{(-4..351)}$ = 0.01289. The response depends on six out of 22 potential predictors (plus the intercept), but the one standard band varies between 6 and 11 predictor variables. The cross-validated R^2 = 0.74. The R package used was glmnet version 4.1-6.[2]

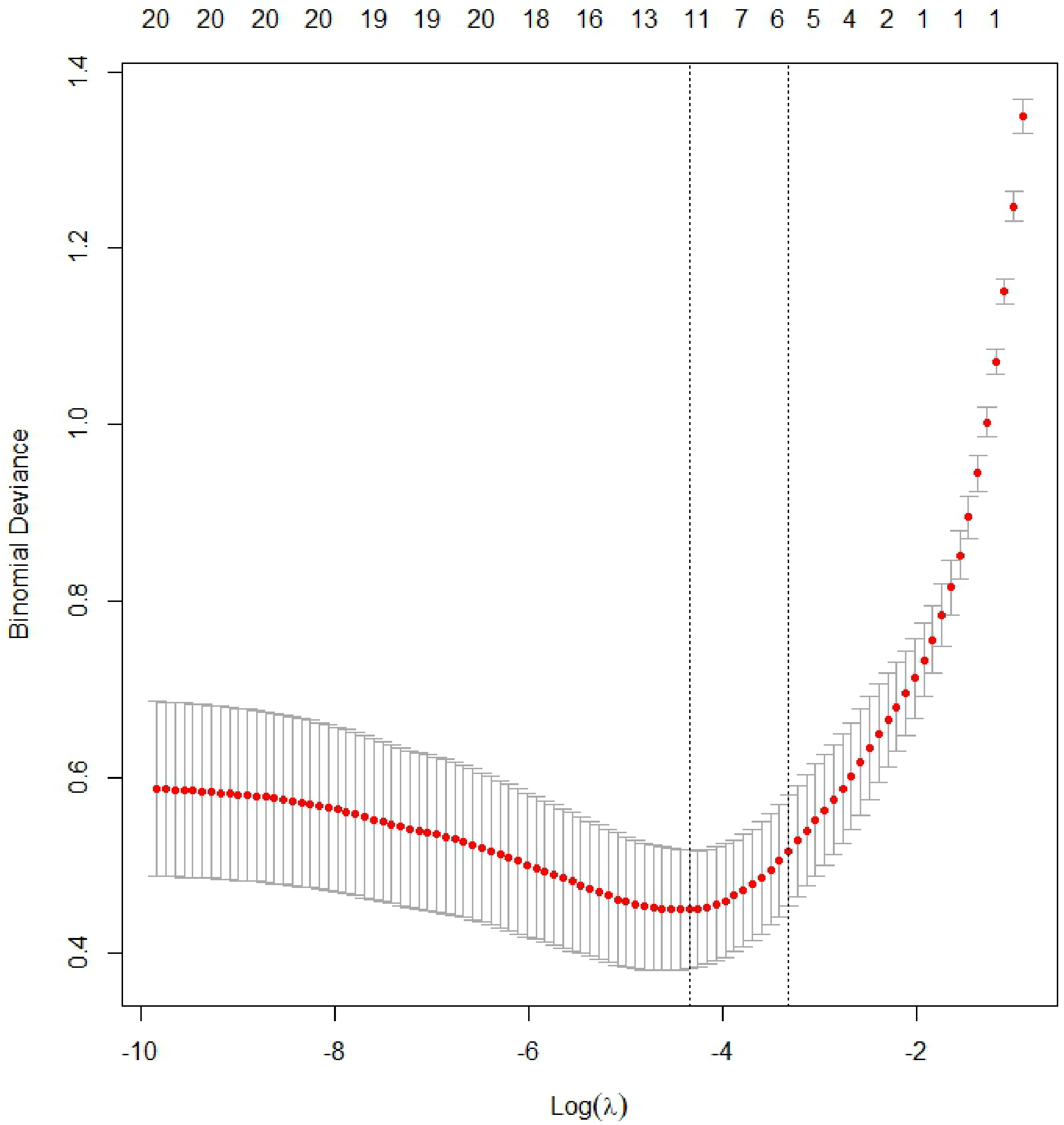

Supplementary Figure S2. Standardized residuals plotted against Leverage in the binary logistic regression model with five predictors (plus intercept).

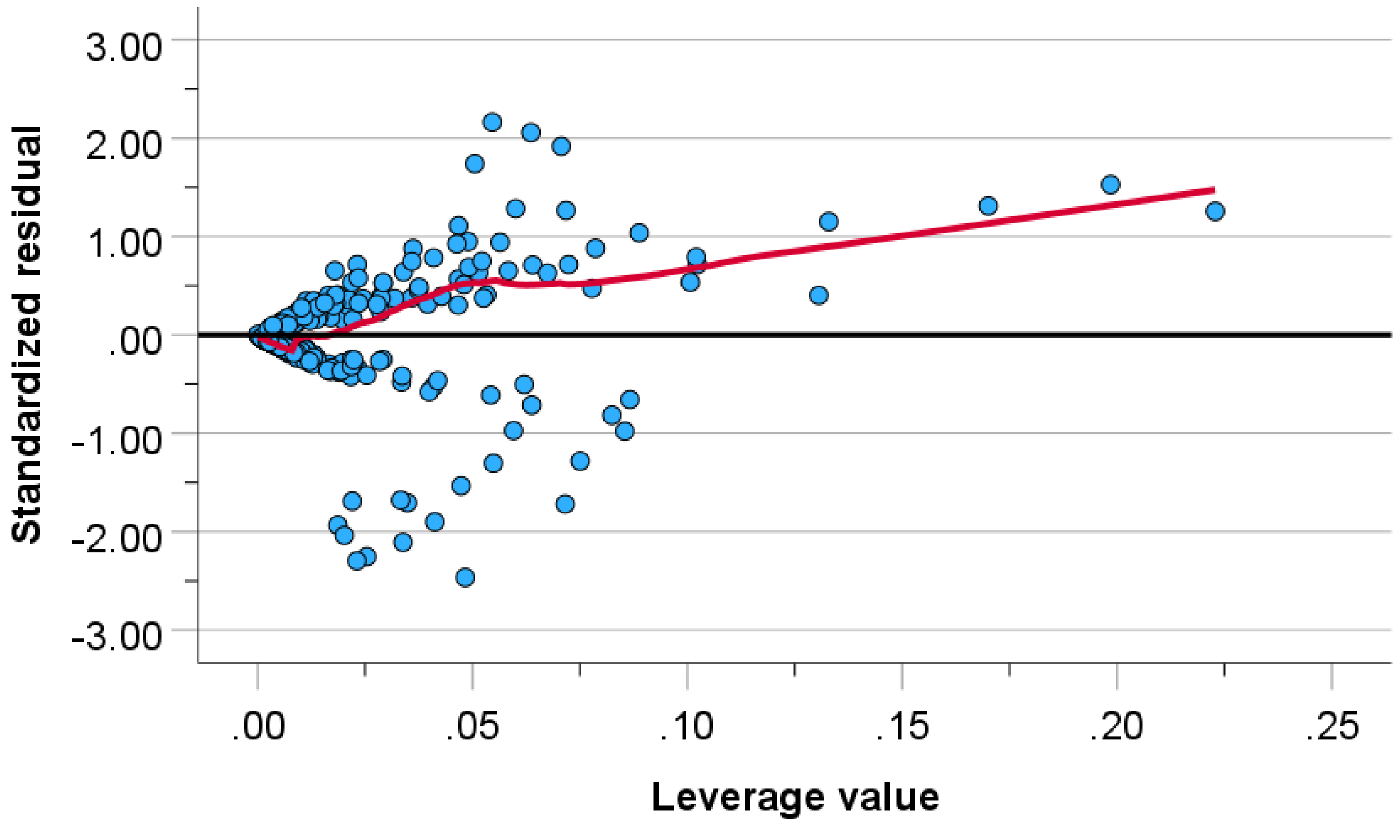

Supplementary Figure S3. Three-dimensional Standardized Residuals plotted against Leverage plotted against Cook's Distance in five predictors (plus intercept).

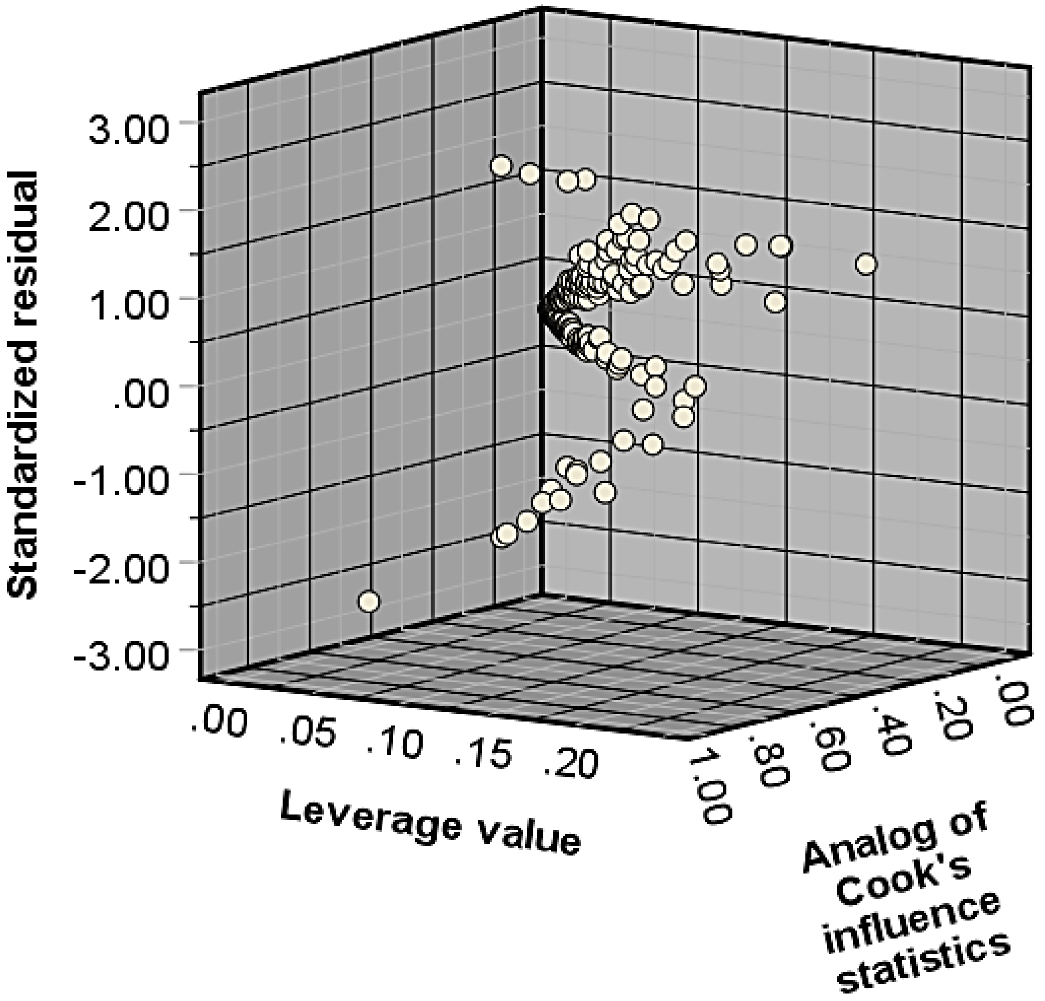

Supplementary Figure S4. Logistic Q-Q (quantile-quantile) plot of standardized residual versus the observed value in the selected binary logistic regression model. This plot assesses the assumption of normality of residuals. The plot reveals the location of the logistic distribution of 0.0019 (mean standardized residuals = 0.0019) and scale equalling 0.36. This indicates that the mean standardized residuals is essentially 0, and that these residuals are relatively tightly distributed around the midpoint, with a relatively small range of values. The scale parameter of 0.36 indicates that the spread of standardized residuals is relatively small and that the assumption of normality of residuals is met for the binary logistic regression model. There is roughly a straight line with points closely following it.

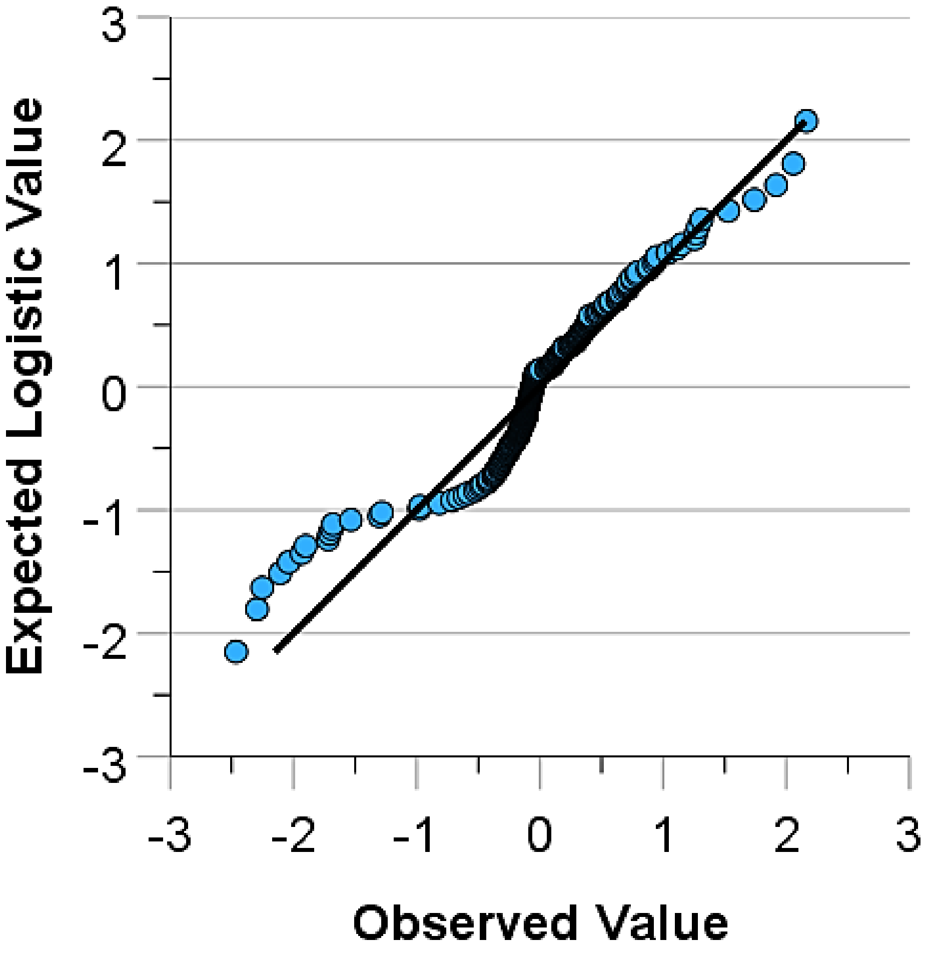

References

1. Friedman J, Hastie T, Tibshirani R. Regularization Paths for Generalized Linear Models via Coordinate Descent. *J Stat Softw.* 2010;33(1):1-22.

2. *Lasso and Elastic-Net Regularized Generalized Linear Models (glmnet R package)* [computer program]. 2022.
